# Direct conversion of N_2_ and O_2_: status, challenge and perspective

**DOI:** 10.1093/nsr/nwac042

**Published:** 2022-03-08

**Authors:** Di Li, Lingxing Zan, Shiming Chen, Zhang-Jie Shi, Ping Chen, Zhenfeng Xi, Dehui Deng

**Affiliations:** State Key Laboratory of Catalysis, Collaborative Innovation Center of Chemistry for Energy Materials, Dalian Institute of Chemical Physics, Chinese Academy of Sciences, Dalian116023, China; State Key Laboratory of Catalysis, Collaborative Innovation Center of Chemistry for Energy Materials, Dalian Institute of Chemical Physics, Chinese Academy of Sciences, Dalian116023, China; State Key Laboratory of Catalysis, Collaborative Innovation Center of Chemistry for Energy Materials, Dalian Institute of Chemical Physics, Chinese Academy of Sciences, Dalian116023, China; Department of Chemistry, Fudan University, Shanghai200433, China; Dalian National Laboratory for Clean Energy, Dalian Institute of Chemical Physics, Chinese Academy of Sciences, Dalian116023, China; Beijing National Laboratory for Molecular Sciences (BNLMS), Key Laboratory of Bioorganic Chemistry and Molecular Engineering of Ministry of Education, College of Chemistry, Peking University, Beijing100871, China; State Key Laboratory of Catalysis, Collaborative Innovation Center of Chemistry for Energy Materials, Dalian Institute of Chemical Physics, Chinese Academy of Sciences, Dalian116023, China

**Keywords:** key components of air, conversion of N_2_ and O_2_, NO_x_ synthesis, C–N–O organics

## Abstract

As key components of air, nitrogen (N_2_) and oxygen (O_2_) are the vital constituents of lives. Synthesis of NO_2_, and C–N–O organics direct from N_2_ and O_2_, rather than from an intermediate NH_3_ (known as the Haber–Bosch process), is tantalizing. However, the extremely strong N≡N triple bond (945 kJ mol^–1^) and the nonpolar stable electron configuration of dinitrogen lead to its conversion being extensively energy demanding. The further selective synthesis of high-value C–N–O organics directly from N_2_, O_2_ and C-containing molecules is attractive yet greatly challenging from both scientific and engineering perspectives. Enormous efforts have been dedicated to the direct conversion of N_2_ and O_2_ via traditional and novel techniques, including thermochemical, plasma, electrochemical, ultrasonic and photochemical conversion. In this review, we aim to provide a thorough comprehension of the status and challenge of the direct conversion of N_2_, O_2_ and C-containing molecules (particularly N_2_ and O_2_). Moreover, we will propose some future perspectives to stimulate more inspiration from the scientific community to tackle the scientific and engineering challenges.

## Teaser

The authors elaborated on the status and challenges of direct conversion of N_2_, O_2_, and C-containing molecules (particularly N_2_ and O_2_) and proposed some future perspectives and development strategies.

## INTRODUCTION

Nitrogen and oxygen, as the key components of air (contributing to 78.1 and 20.9 vol.%, respectively), are the vital constituents of living organisms and the critical elements of proteins, DNA, RNA and amino acids [1,2]. Humans and animals require some particular amino acids every day for nutrition and survival [3]. On the other hand, nitrogen compounds are essential for the growth of plants and they can even synthesize amino acids from these nitrogen sources. Regarding the non-biological aspect, nitrogen oxides (NO_x_) are a crucial building stock for chemistry and industry. Their further products, ranging from nitric acid (HNO_3_), carbamide (CH_4_N_2_O) to adiponitrile (NC(CH_2_)_4_CN), are highly desired and widely used in fertilizer and plastic manufacturing, etc. For example, the world's annual production of HNO_3_ reached 67.8 million tons in 2017 [4], while the demand for HNO_3_ products is still increasing [4]. By far, almost 80% of produced nitric acid is used in the manufacture of fertilizers, among those 96% is used to produce ammonium nitrate and calcium ammonium nitrate. Others can be used to produce intermediates in the polymer industry, particularly in the manufacture of adipic acid to produce polyamides, toluene diisocyanate to produce polyurethanes and nitrobenzene to produce dyes [4]. However, being the most abundant source in the atmosphere, nitrogen is elusive for almost all living organisms (except diazotrophs) due to its inertness. The extremely strong triple bond (945 kJ mol^–1^) and nonpolar stable electron configuration lead to its chemical conversion being extensively energy demanding. Thus, the naturally abundant nitrogen must first be manually ‘fixed’ (so-called nitrogen fixation).

In nature, some micro-organisms can capture the atmospheric nitrogen and supply it to plants as biological nitrogen fixation [5] and it annually contributes to ∼297 million metric tons of fixed nitrogen [6]. Apart from biological nitrogen fixation, oxidation of N_2_ to NO_x_ can occur through lightning formed from the electric discharge between two clouds or between clouds and the earth if the conditions (pressure or temperature) are proper for the formation of NO_x_. This process, as a combination of heat shock with electric discharge (thermal and plasma process), represents the early natural nitrogen fixation into NO_x_ in Earth's atmosphere [6]. The total natural non-biological nitrogen fixation is reported at ∼171 million metric tons per year [7]. Besides, human activity, such as the combustion of fuels, can more or less contribute to nitrogen fixation. Yet, it takes responsibility for some environmental issues as unusable NO_x_ is undoubtedly a pollutant in the atmosphere [8]. The global population expansion has intensified the demand for usable nitrogen sources not only in terms of agriculture, where nitrogen from the soil is diminishing quickly, but also in terms of industry. The naturally fixed nitrogen cycle is too slow and uncontrollable to satisfy the growing demand for nitrogen sources.

**Figure 1.**
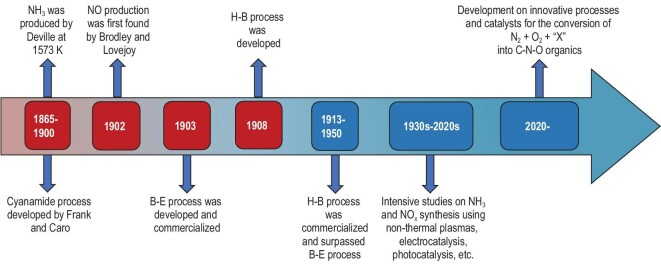
The timeline of milestones in the chemical nitrogen-fixation process, adapted from Refs [9,17–19].

At the beginning of the twentieth century, scientists devoted lots of effort to fixing atmospheric nitrogen as the natural process had no longer met the demands. In 1895–98, German scientists Frank and Caro developed the Frank–Caro cyanamide process. In this process, nitrogen was fixed in the form of calcium cyanamide by the reaction of calcium carbide with nitrogen [9]. The first industrialized application of atmospheric nitrogen fixation mimicked the natural process of lightening, as we have mentioned previously. This process, known as the Birkeland–Eyde (B–E) process, directly produces NO_x_ from atmospheric nitrogen and oxygen using electric arcs to induce high temperature, whereby the activation of N_2_ molecules can be triggered [10]. The B–E process was successfully realized and reported in 1903, and so was the first successfully industrialized plasma process [11]. The plasma arcs were generated in the B–E furnace, where the temperature was high due to the thermal plasma heating, then the air rapidly passed the furnace to ‘combust’ and produce NO_x_. However, the energy efficiency of this process was inconsiderable and later it was prevailed by a more practical process, known as the Haber–Bosch (H–B) process. The H–B process, which was first developed in 1908 and commercialized in 1913, produces ammonia by initiating the reaction of pure nitrogen and hydrogen under heating and pressurizing conditions with the presence of iron-based catalysts [12]. It was soon extensively studied and widely applied in industries. Over recent decades, significant development in engineering and fundamental aspects has been achieved to marginally reduce the energy consumption of the H–B process and comprehend the reaction mechanism [13]. Until now, the H–B process has contributed to nearly 50% of the nitrogen found in human tissues and feeds ∼40% of the world's population [14]. Nevertheless, this process suffers from extreme conditions (heating and high pressure). It also consumes a large amount of pure hydrogen, which was unavailable in nature and has to be obtained from a steam reforming process, generating ∼1.9 metric tons of CO_2_ per metric ton of NH_3_ production (3 CO_2_ per 8 NH_3_) [15]. On the other hand, the produced NH_3_ is still an intermediate in the industrial nitrogen source cycle, used as the feedstock for further products such as HNO_3_ through an Ostwald process. Compared to the H–B process, a more desirable route of N_2_ fixation is the direct conversion of N_2_ into N-containing organic compounds under mild conditions [16]. The timeline of milestones in the chemical nitrogen-fixation process is shown in Fig. 1 for convenience [9,17–19].

**Figure 2.**
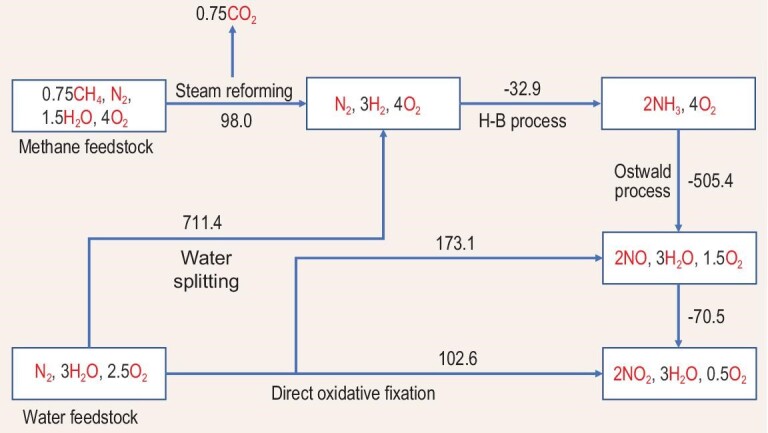
Reductive and oxidative N_2_-fixation pathways with energy expenditures. The values along the arrow lines are the standard Gibbs free energies in kJ mol^–1^ of fixed N_2_, adapted from Refs [2,21].

**Scheme 1.**
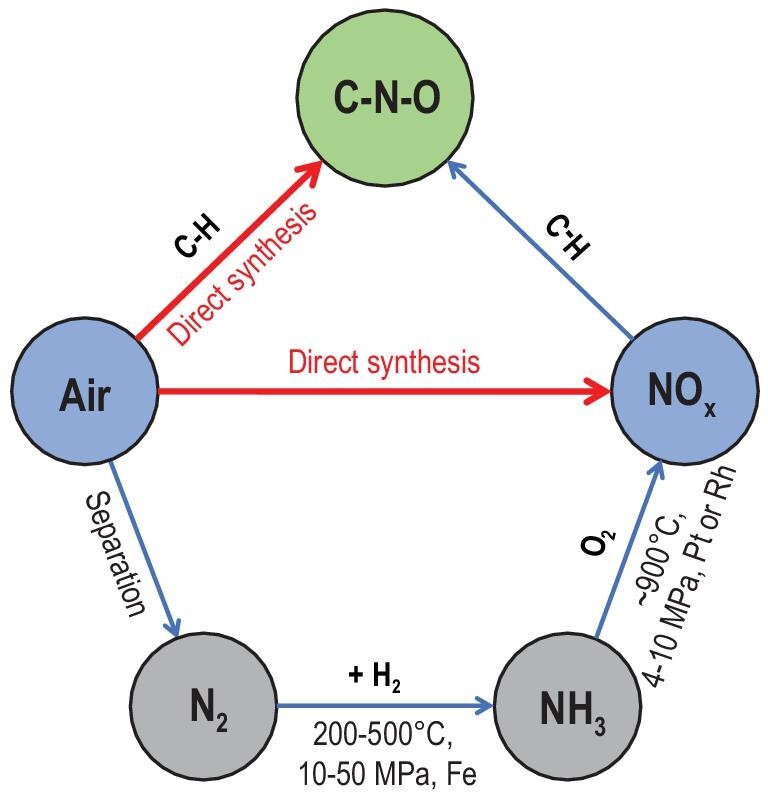
The conversion pathways of key components of air.

The energy expenditures for various reductive and oxidative N_2_ fixation pathways have been well concluded and illustrated by Chen *et al.* [2,20]. We simplified the completed data and illustrated them in Fig. 2. HNO_3_ is typically produced through the three-step process of steam reforming followed by the H–B process, then the Ostwald process for NO_2_ production. However, as aforementioned, the overall process is exceptionally energetically extensive and the emission of CO_2_ is also dramatic. The improvement in the reaction process may alleviate the energy consumption to some extent; for example, the development of catalysts could conduct the steam reforming or H–B process under milder conditions such as avoiding pressurizing. Nevertheless, the direct oxidative process is more attractive in terms of theoretical energy efficiency, enrichment of feedstock (two main air components) and the convenience for engineering design if it can be successfully realized. The B–E process has demonstrated the possibility for the direct oxidative conversion of main air components. However, the present energy consumption of this thermal plasma process is far more than that of the three-step process, not to mention the other drawbacks such as the high investment for the equipment and the decomposition of NO_x_. In principle, the oxidative conversion of N_2_ with O_2_ can undergo a lower energy input than the three-step process, which indicates great potential to replace the three-step process. The fundamental chemistry of the reaction merits extensive investigation. Meanwhile, since many C–N–O organics are highly required, introducing a carbon-containing molecule ‘X’—either a simple gas or complex organic in this oxidation and conversion of the N_2_ process to synthesize C–N–O products such as amino acids—is of great significance. The scheme of the conversion pathways is represented in Scheme 1.

Herein, we will elaborate on the status and the challenges on the conversion of N_2_ and O_2_, and address the future perspectives of the conversion of N_2_/O_2_/‘X’ into high-value products. Furthermore, as the direct conversion of N_2_ must overcome an enormous-amount-of-energy barrier, thus demanding extreme temperatures, this review will mainly focus on the works and possibilities to realize this process under soft conditions, such as non-thermal plasma, electrochemical, ultrasonic and photon-driven conversion. The representative works are summarized in Table 1. All these points will be carefully illustrated in the following sections.

**Table 1.** Representative work on non-thermal plasma, electrochemical, ultrasonic and photon-driven conversion of N_2_, O_2_ and ‘X’.

Process	Parameter	Catalyst	Condition	Production	Yields	Ref.
Plasma	Radio frequency: 2.6–3.2 MHz; Power: 150 W	None	100–350°C	HNO_3_	2.9–9.4 g kWh^–1^	[27]
	Plasma jet: 1.3–3.1 kV; Power: 0.6–26.27 W	None	35 ± 5°C	NO and NO_2_	NO: 750–1050 ppm at maximum; NO_2_: negligible	[29]
	DC pulsed discharge: 1–10 kHz; power: 0–6 W	None	∼ 3000°C	NO and NO_2_	NO_x_ generation rate: ∼7 × 10^16^ J^–1^	[28]
	Microwave: 2.48 GHz; power: 30–90 W; water added	None	2000 Pa	NO, N_2_O, NO_2_, NO_3_, HNO_2_ and HNO_3_	Not quantify	[31]
	DC gliding arc: 0–100 W; current: 10–80 mA	Al_2_O_3_	1400 ± 300°C	NO and NO_2_	NO: 3117 ppm; NO_2_: 213 ppm	[32]
	Rotating gliding arc; power: 108 and 200 W; voltage: 2 and 3 kV	None	1700 and 2300–2700°C	NO_x_	NOx concentrations up to 5.5%	[36]
	DBD; power: 45 W; frequency: 18 kHz	5% of WO_3_, PbO, CuO, Co_3_O_4_, MoO_3_, NiO or V_2_O_5_ on γ- Al_2_O_3_	Less than 200°C	NO_x_	NOx concentrations up to 5700 ppm	[37]
	DBD; energy density: 777 J L^–1^; frequency: 480 Hz; water added	None	25–325°C	NO_x_, HNO_2_ and HNO_3_	NO_2_: 250 ppm at maximum; N_2_O: 58.8 ppm at maximum	[38]
	DBD; power: 15–72 W; voltage: ∼9 kV; frequency: 10 kHz; water added	None	25–600°C	NH_4_^+^, NO_2_^–^ and NO_3_^–^	NH_3_: 10.8 ± 1 μmol h^–1^; NO_2_^–^: 10 ± 2 μmol h^–1^; NO_3_^–^: 43 ± 4 μmol h^–1^	[41]
Ultrasound	Frequency: 30 kHz; power density: 10 W cm^–2^	None	Water, pH was adjusted by sulfuric acid (1–3); by acetate buffer (3–5); by phosphate buffer (5–8); phosphate and sodium hydroxide (>8), 18–20°C	NO_2_^–^ and NO_3_^–^	NO_2_^–^: ∼191 μmol L^–1^ h^–1^ (pH = 11.2); NO_3_^–^: ∼168 μmol L^–1^ h^–1^ (pH = 1.25)	[69,70]
	Frequency: 447 kHz; power: 50 W	None	H_2_O, 25 ± 0.2°C	NO_2_^–^ and NO_3_^–^	NO_2_^–^: 172 μmol L^–1^ h^–1^; NO_3_^–^: 26.4 μmol L^–1^ h^–1^	[71]
	Frequency: 35 kHz; power: not mentioned	None	H_2_O, 20°C	NO_2_^–^ and NO_3_^–^	NO_2_^–^: 16 μmol L^–1^ h^–1^; NO_3_^–^: 5 μmol L^–1^ h^–1^	[73]
	Frequency: 900 kHz; power: 27 W in 100 mL H_2_O	None	H_2_O, ∼5°C	NO_x_^–^	NO_x_^–^: 486 μmol L^–1^ h^–1^	[74]
	Frequency: 28 kHz; power: 1200 W	None	H_2_O, 25°C	NO_2_^–^ and NO_3_^–^	NO_2_^–^: 36.15 μmol L^–1^ h^–1^; NO_3_^–^: 26.4 μmol L^–1^ h^–1^	[19]
Electrocatalysis	2.19 V vs. RHE	Pt foil	0.3 M K_2_SO_4_, room temperature	NO_2_^–^ and NO_3_^–^	NO_2_^–^: 0.0004 μmol h^–1^ cm^–2^; NO_3_^–^: 0.06 μmol h^–1^ cm^–2^	[55]
	1.96 V vs. RHE	Fe-SnO_2_	0.05 M H_2_SO_4_, room temperature	NO_3_^–^	NO_3_^–^: 42.9 μg h^–1^ mg_cat._^–1^	[58]
	2.03 V vs. RHE (J = 0.4 mA cm^–2^)	Pd-MXene (Ti_3_C_2_T_x_, T = F and O)	0.01 M Na_2_SO_4_, room temperature	NO_3_^–^	NO_3_^–^: 2.8 μg h^–1^ mg_cat._^–1^	[59]
	2.2 V vs. RHE	Ru(2.79 wt%)/TiO_2_	0.1 M Na_2_SO_4_, room temperature	NO_3_^–^	NO_3_^–^: 161.9 μmol h^–1^ mg_cat._^–1^	[56]
	1.6 V vs. RHE	ZnFe_0.4_Co _1.6_O_4_	1 M KOH, room temperature	NO_3_^–^	NO_3_^–^: 130 ± 12 μmol h^–1^ g _MO_^–1^	[57]
Photocatalysis	UV irradiation (4.81 ± 0.51 mW cm^–2^ at 253.7 nm)	TiO_2_	H_2_O, 25 ± 0.1°C	NO_3_^–^	NO_3_^–^: 3.51 ± 0.06 mg h^–1^ m^–2^	[86]
	UV irradiation (100 mW cm^–2^ at 365 nm)	TiO_2_/WO_3_	200–350°C	NO	NO: 0.16 mmol g^–1^ h^–1^ (300°C)	[88]
	UV irradiation (1.5 Mw cm^–2^ at 380 nm)	Pothole-rich WO_3_	H_2_O, room temperature	NO_3_^–^	NO_3_^–^: 1.92 mg g^–1^ h^–1^	[89]

## N_2_ + O_2_

### Thermochemical conversion

At first, we will discuss the direct oxidation of N_2_ by O_2_. NO_x_ can be generated during the combustion process in the air when the temperatures are >1473 K. NO is the primary oxidative component of NO_x_, which can be addressed by the following reaction:

(1) \begin{equation*}{{\rm{N}}_2} + {{\rm{O}}_2}{\rm{\ }} \leftrightarrow 2{\rm{NO}}\end{equation*}

The standard enthalpy of NO formation is 90.3 kJ mol^–1^ NO, indicating that the positive reaction is unfavorable under low temperatures. This reaction has a negligible rate even at combustion temperatures. Zeldovich proposed that NO is produced during combustion via radical reactions, where N_2_ or O_2_ dissociates into N or O radicals. The process can be illustrated as Equations 2 and 3 [21]:

(2) \begin{equation*}{\rm{O}}\! \bullet +\ {{\rm{N}}_2}{\rm{\ }} \to {\rm{NO}} + {\rm{\ N}} \!\bullet \end{equation*}

(3) \begin{equation*}{\rm{N}} \!\bullet +\ {{\rm{O}}_2}{\rm{\ }} \to {\rm{NO}} + {\rm{O}} \!\bullet \end{equation*}

**Figure 3.**
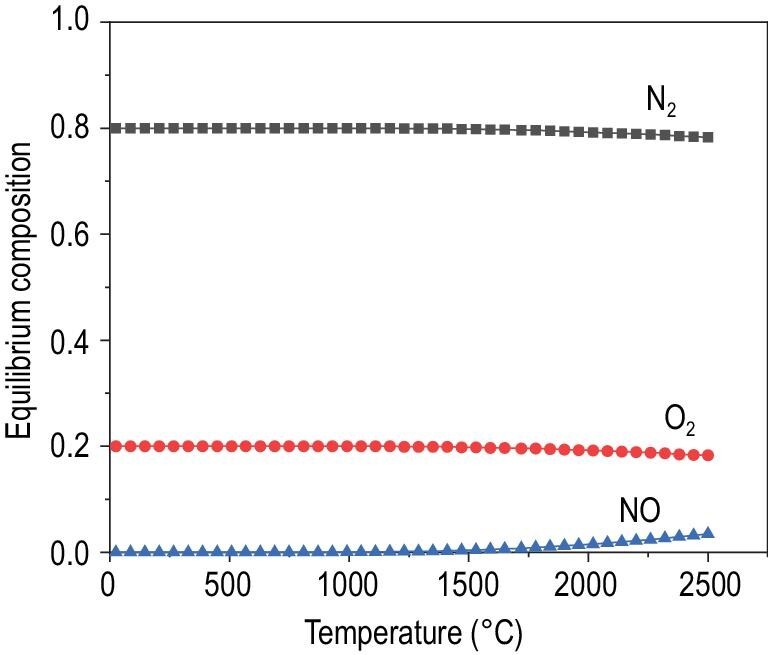
Equilibrium composition of NO, calculated using HSC Chemistry v6.0.

We calculated the equilibrium composition of NO_x_ using HSC Chemistry v6.0. The results (Fig. 3) show that the equilibrium NO concentration reaches only 3.4% in the air at 2500°C while the other NO_x_ products are negligible. Thus, the reaction is tough to operate via a typical thermochemical process due to the demand for high temperature and the difficulty in developing catalysts. The catalyst needs to remain stable and activate N_2_ with the presence of O_2_ at these extreme temperatures. Unless an efficient catalyst can initiate the reaction under milder temperatures with pressurizing, the process is, for now, not suitable to conduct under thermochemical conditions.

The analogous radical reactions also occur in the thermal plasma process, such as in the B–E process. The gas molecules are highly dissociated and ionized in thermal plasmas through the plasma arcs under high temperatures. Similarly, catalysts are also impossible to pack into a thermal plasma reactor due to the extreme conditions (several thousand K and plasma arcs), where the catalysts are probably out of work function or melted, corroded and decomposed. Moreover, the B–E process for conversion (3.4–4.1 MJ mol^–1^ HNO_3_ production) [22] is far more energy wasteful than the H–B process. The overall energy efficiency of N_2_ activation in thermal plasmas is inconsiderable and the dissociation of NO_x_ products is competitive with the forward reaction when no thermal quenching exists. Due to these limitations, the research based on thermal conditions has not progressed much yet. Alternative methods are essential to compensate for the energy requirements for the direct oxidation of N_2_ by O_2_ under mild conditions. For these reasons, electro-, photo-, ultrasonic- or non-thermal plasma-assisted conversion routes probably prevail and offer more excellent opportunities for progress. Moreover, the reasonable combination of two or more of those techniques may bring out ‘magical’ synergies, achieving green and mild N_2_ fixation.

### Non-thermal plasma conversion

**Figure 4.**
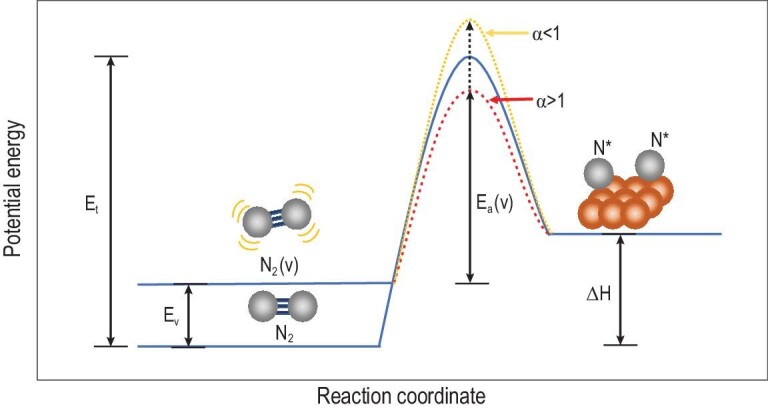
Reaction coordinate of activation energies for N_2_ dissociation starting from ground (blue) or vibrationally excited states. The dashed red and yellow curves correspond to different vibrational efficiency (α), adapted from Ref. [23].

Non-thermal plasmas (NTPs) are a feasible solution to alleviate the limitations owing to their unique non-equilibrium property. A non-thermal plasma consists of high-energy electrons, excited species and ions. The temperature of high-energy electrons can exceed several thousand K while the bulk temperature can remain ambient. As a result, the thermally inaccessible chemical reactions can be triggered under mild conditions, thus potentially coupling with catalysis. The direct dissociation of N_2_ is difficult in NTPs as the dissociation energy is 9.8 eV for its triple bond, while the mean electron energy is typically ∼1–3 eV in NTPs. Therefore, the vibrationally excited N_2_, as the most atmospherically enriched species, plays an essential role in the NTP process. On the one hand, the accumulation of vibrational quanta from several vibrationally excited N_2_ molecules into one could lead to high vibrational quanta, and thus direct dissociation of the acceptor. On the other hand, a more efficient dissociative adsorption can occur on the catalysts, as shown in Fig. 4 [23]. The dissociation of a ground-state N_2_ molecule undergoes the path with activation energy *E*_t_ in the energy-potential diagram. For a vibrationally excited N_2_ molecule, the energy of the initial state is increased by the energy of vibration *E*_v_. The vibrationally excited N_2_ molecule can either undergo an ideal path, where the enhancement of the initial state energy decreases the dissociation barrier by a value equivalent to (blue curve) or more than *E*_v_ (red curve), or undergo a general path, where the vibrational coordinate does not project on the reaction coordinate (yellow curve) [24]. The single, extended vibrational ladder and a relatively large vibrational spacing (∼0.3 eV) of the N_2_ molecule make the enhancements in the dissociation rates possible through vibrational excitation [23]. According to current approximates, theoretical energy consumption for the conversion of N_2_ with O_2_ into NO production via Equation (1) in a non-thermal plasma (∼400 kJ mol^–1^ N_2_) is >2.5 times lower than that for the H–B process with a methane-derived H_2_ source. Moreover, the energy efficiency already achieved in the laboratory scale, which ranges from 600 to 1200 kJ mol^–1^ N_2_ assuming 100%-efficient plasma generation [25], is far better than the H–B process (∼3000 kJ mol^–1^ N_2_ consuming H_2_ from water electrolysis) [26]. If the energy consumption can be reduced to 1000 kJ mol^–1^, the NTP process will prevail over the three-step processes.

Indeed, from the 1980s, intensive studies on NTPs assisted conversion of N_2_ have been conducted, including different NTP types such as radiofrequency discharges [27], DC discharge [28,29], microwave discharge [30,31], glow discharge [32,33], pulsed arc and gliding arc discharge [34–36], dielectric barrier discharge (DBD) [37–41] and some related modeling [36,42–44]. Among those, DBD has drawn great interest due to its simple design and convenience for industrialization and integration with catalysts. For example, Patil *et al.* studied the direct synthesis of NO_x_ by packing different catalyst support materials in a DBD reactor [37]. Different surface areas, relative dielectric constants and particle shapes due to the different properties of support materials and their particle sizes had a significant effect on the formation of NO_x_ [37]. However, the author also proposed that the non-catalytic route via direct gas-phase interaction of excited N_2_ with O_2_ species was dominant [37]. Other information can be found in the publications mentioned above.

As high-energy species can be effectively generated in NTPs, the generation of vibrationally excited N_2_ species can be written as [45]:

(4) \begin{equation*}{{\rm{e}}^ - } + {{\rm{N}}_2}{\rm{\ }} \to {{\rm{e}}^ - } + {\rm{\ }}{{\rm{N}}_2}\!\left( {\rm{v}} \right)\end{equation*}

Then NTPs could accelerate the Zeldovich-like reactions in the plasma atmosphere, which can be depicted as [25,35]:

(5) \begin{equation*}{{\rm{N}}_2}\!\left( {\rm{v}} \right) + {\rm{O}} \!\bullet {\rm{\ }} \to {\rm{NO}} + {\rm{\ N}}\left( {\rm{v}} \right) \!\bullet \end{equation*}

(6) \begin{equation*}{\rm{N}}\left( {\rm{v}} \right) \!\bullet +\ {{\rm{O}}_2}{\rm{\ }} \to {\rm{NO}} + {\rm{\ O}} \!\bullet \end{equation*}

While in the presence of a catalyst, the dissociative adsorption of a vibrationally excited N_2_(v) molecule promoted by NTPs existed in parallel and then the mobile oxygen reacts with the adsorbed N on the surface to give away NO. The reaction of adsorbed nitrogen with mobile oxygen was found to be rate-determining [46]:

(7) \begin{equation*}{{\rm{N}}_2}\!\left( {\rm{v}} \right)\mathop {\Longleftrightarrow} \limits^{{\rm{surface}}} 2{{\rm{N}}^{\rm{*}}}\end{equation*}

(8) \begin{equation*}{\rm{O}} + {{\rm{N}}^{\rm{*}}}\mathop {\Longleftrightarrow} \limits^{{\rm{surface}}} {\rm{N}}{{\rm{O}}^{\rm{*}}}\mathop {\Longleftrightarrow} \limits^{{\rm{desorption}}} {\rm{NO}}\end{equation*}

Electronic excitation can also lead to the acceleration of NO_x_ production while being limited by high energy costs and low efficiency [25]. MoO_3_ and WO_3_ seem to be effective catalysts in this process [37]. However, experimental results indicate that plasma atmospheric reactions and plasma–surface reactions occur in parallel, leading to a more complex work function of catalysts in plasma [37]. The understanding of complex plasma–catalyst synergistic effects is indeed of great importance.

Other types of NTPs and the related engineering optimization have also been widely studied for NO_x_ productions. For example, Jardali *et al.* [36] developed an atmospheric-pressure rotating gliding arc plasma reactor for highly efficient NO_x_ production (concentration of ≤5.5%). The authors also studied the behavior of the plasma arc using various numerical modeling patterns. Their experimental and modeling results indicated that both the vibrationally promoted and the Zeldovich mechanisms dominated in the plasma zone as the gas and vibrational temperatures are in equilibrium at ∼2600 K [36]. It should be noticed that catalysis is inconvenient to couple in these plasma processes, thus the selectivity towards one product, such as NO_2_, is beyond control. In fact, even for a plasma-catalytical process in a DBD reactor, the selectivity to NO_2_ is unsatisfactory. There is a pressing need to comprehend the fundamental mechanisms that unite plasma physical chemistry, gas–surface chemistry and catalysis to guide the rational design of a plasma reactor, and thus the optimization of plasma type and the development of packed-catalytic materials.

### Electrochemical conversion

Direct oxidation of nitrogen to nitrogen-containing compounds such as oxynitride, nitrite and nitrate is one of the most challenging reactions in electrochemistry. According to the standard electrode potentials for the conceivable half-reactions calculated from their thermodynamic properties [47,48], useful oxidation state diagrams plotted with the volt equivalent of the half-reaction of a particular nitrogen compound to nitrogen versus its oxidation state were presented in the literature [49]. Dinitrogen and ammonia are suggested to be the most stable under standard conditions and a steep climb for the oxidation of nitrogen indicates that extraordinarily high energy is essential [49].

The electrochemical reaction process involves reactant dissolution, mass transportation, adsorption, reaction and desorption steps, and is always accompanied by the decomposition of a solvent such as hydrogen evolution, oxygen reduction and oxygen evolution reactions in aqueous solution aroused by the competitive adsorption. However, except for the inherent inertia characteristic of nitrogen, the lower solubility, weaker adsorption and higher activation energy, which are strongly associated with the properties of reactants and solutions, electrode potential and the catalytic activity of the electrode material also make it extremely difficult for the electrochemical oxidation reaction of nitrogen to be achieved. As a consequence, only a few studies on this topic have been reported so far.

The electrode potential and the electrolyte play important roles in an electrochemical reaction and should be selected and optimized reasonably based on empirical evidence and in-depth understanding. According to the Nernst equation, the standard potentials versus standard hydrogen electrode of the oxygen evolution reaction (OER) and nitrogen oxidation reaction (NOR) at 298 K and 1 atm were calculated to be *E*^θ^ = +1.23 V and *E*^θ^ = +1.24 V, respectively, which are very close to each other. Fundamental studies have been launched [50,51] and the potential-pH partial Pourbaix diagram for the N_2_–H_2_O system was established [2]. Above the line of N_2_ oxidation, it is possible to oxidize dinitrogen directly towards NO_3_^–^ under moderately oxidizing conditions. As is well known, the OER is inevitable during the NOR in aqueous electrolytes. Hence, the selections of solution pH and electrode potential become vitally essential to control the competition process of oxygen evolution appropriately. Besides, the detailed information on potential and pH-dependent electrochemically stable nitrogen species was presented in this diagram. It was proposed from the thermodynamic point of view that NOR via a 10-electron-transfer process is more favorable than the four-electron process of OER at pH > 1.3 (Equation 10), particularly in neutral and alkaline electrolytes. An attractive alternative electrochemical route to HNO_3_ is via a redox reaction (Equation 11) in which the standard Gibbs free energy was estimated to be 14.6 kJ mol^–1^ N_2_ [51]. Consequently, NOR is not as easy as we anticipated and a sufficiently active and selective electrocatalyst is indispensable for triggering this reaction:

(9) \begin{equation*}{{\rm{O}}_{2({\rm{g}})}} + 4{{\rm{H}}^ + } + 4{{\rm{e}}^ - } \mathbin{\lower.3ex\hbox{$\buildrel\textstyle\rightarrow\over {\smash{\leftarrow}\vphantom{_{\vbox to.5ex{\vss}}}}$}} {\rm{ }}2{{\rm{H}}_2}{\rm{O}}\end{equation*}

(10) \begin{equation*}{{\rm{N}}_{2({\rm{g}})}} + 6{{\rm{H}}_2}{{\rm{O}}_{({\rm{liq}})}} \mathbin{\lower.3ex\hbox{$\buildrel\textstyle\rightarrow\over {\smash{\leftarrow}\vphantom{_{\vbox to.5ex{\vss}}}}$}} 2{\rm{N}}{{\rm{O}}_3}{^ - \!_{\left( {{\rm{aq}}} \right)}} + 12{{\rm{H}}^ + }_{({\rm{aq}})} + 10{{\rm{e}}^ - }\end{equation*}

(11) \begin{eqnarray*}{{\rm{N}}_{2({\rm{g}})}} && + \ 2.5{{\rm{O}}_{2({\rm{g}})}} +\ {{\rm{H}}_2}{{\rm{O}}_{({\rm{liq}})}} \mathbin{\lower.3ex\hbox{$\buildrel\textstyle\rightarrow\over {\smash{\leftarrow}\vphantom{_{\vbox to.5ex{\vss}}}}$}} 2{{\rm{H}}^ + }_{({\rm{aq}})}\\ &&+\, 2{\rm{N}}{{\rm{O}}_3}{^ -\! _{\left( {{\rm{aq}}} \right)}}\end{eqnarray*}

Requirements of a rational catalyst design for NOR should be far stricter than those for OER, oxygen reduction reaction and hydrogen evolution reaction, because it needs to be sufficiently active and accurately balance the competitive adsorption, activation and dissociation of N_2_ and H_2_O/OH^–^ and the desorption of products processes. In principle, the reaction rate is measured by the activation energy for N_2_ dissociation, which determines the rate of dissociation and/or the effective adsorption of nitrogen and is limited by the activity and number of active sites on the surface. For reductive nitrogen fixation to ammonia, the dissociation of N_2_ as the rate-determining step on the most active catalysts was proposed by both theoretical calculation and experiments [52–54]. Analogously for NOR, the rate-determining step was also determined to be the dissociation of N_2_. The challenging task should be undertaken by developing highly efficient catalysts.

**Figure 5.**
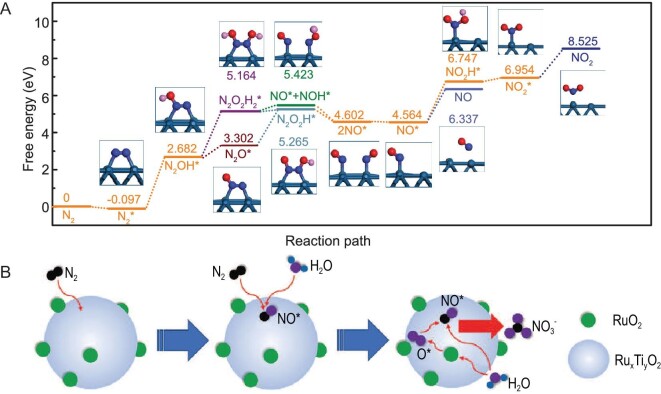
(A) Calculated free-energy diagram for N_2_ electrooxidation over platinum foil. Blue ball (N atom), red ball (O atom), pink ball (H atom) and green ball (Pt atom), reproduced with permission from Ref. [55]. Copyright 2019, Oxford University Press. (B) The NOR mechanism of Ru/TiO_2_ composite electrocatalysts, reproduced with permission from Ref. [56]. Copyright 2020, Wiley-VCH.

Precious metals with distinct chemical and physical properties such as having not fully filled d-orbitals and having moderate adsorption strength, easy adsorption reactants and formation intermediates that result in a strong performance of catalytic activity and stability are highly preferred in the field of electrocatalysis. To our knowledge, the initial work that centered on NOR was carried out on a Pt electrode surface by Zhang *et al.* [55]. Direct electrocatalytic oxidation of N_2_ to HNO_3_ was successfully achieved in the electrolyte of 0.3 M K_2_SO_4_ saturated with air as the nitrogen source by applying a potential of +2.19 V versus reversible hydrogen electrode (RHE), where the Faradaic efficiency was calculated to be only ∼1.23%. The yields of NO_3_^–^ and NO_2_^–^ reached 0.06 and 0.0004 μmol h^–1^ cm^–2^, respectively. With a further increase in the applied potential, the yields remained almost unchanged. Density-functional theory (DFT) calculations suggested that the NOR was through multiple processes as shown in Fig. 5A. The initial step is the adsorption of the N_2_ molecule to form N_2_^*^ on the platinum surface spontaneously, closely followed by the sluggish step of adsorbed N_2_^*^ with OH^–^ to produce N_2_OH^*^, which is then dehydrogenated to N_2_O^*^ or probably undergoes a thermodynamically unfavorable approach with OH^–^ to form N_2_O_2_H_2_^*^. Then through a transition state, both N_2_O^*^ and N_2_O_2_H_2_^*^ can be evolved into the formation of NO^*^. NO^*^ may undergo the desorption step from the Pt surface and be oxidized into HNO_3_ and HNO_2_ in the electrolyte (Equation 12). Alternatively, NO^*^ can also be oxidized into NO_2_^*^ at the Pt surface and then desorbed with a further increase in potential and transformed into HNO_3_ in the electrolyte (Equation 13):

(12) \begin{equation*}2{\rm{N}}{{\rm{O}}_{({\rm{g}})}}\! + {{\rm{H}}_2}{{\rm{O}}_{({\rm{liq}})}} \!+ {{\rm{O}}_{2({\rm{g}})}} \to {\rm{HN}}{{\rm{O}}_{3({\rm{aq}})}} + {\rm{HN}}{{\rm{O}}_{2({\rm{aq}})}}\end{equation*}

(13) \begin{equation*}2{\rm{N}}{{\rm{O}}_{2({\rm{g}})}} + {{\rm{H}}_2}{{\rm{O}}_{({\rm{liq}})}} + 1/2{{\rm{O}}_{2({\rm{g}})}} \to 2{\rm{HN}}{{\rm{O}}_{3({\rm{aq}})}}\end{equation*}

Interestingly, the synergistic/bifunctional catalytic effect was found on the catalysts consisting of metals and metal oxides, and the catalytic performances may compete with or even surpass those of precious metals. Recently, Yan *et al.* [56] revealed that well-designed Ru-doped TiO_2_/RuO_2_ performed an efficient synergistic catalytic activity towards NOR in a 0.1 M Na_2_SO_4_ electrolyte saturated with pure nitrogen under ambient conditions. After optimizing the experimental conditions and catalyst composition, at a 2.79(wt%)Ru/TiO_2_ catalyst surface, the highest yield rate of 161.9 μmol h^–1^ g_cat._^–1^ and highest Faradaic efficiency of 26.1% were achieved at potentials of 2.2 and 1.8 V (versus RHE), respectively. It was speculated that two steps probably evolved in this reaction as depicted in Fig. 5B. The first step, which was considered as the rate-determining step, is NO^*^ intermediate formation by N_2_ electrochemical activation and oxidation on Ru_x_Ti_y_O_2_ (Equations 14–18), where the OER competing side reaction was suppressed; the second step is nitrate formation by non-electrochemical oxidation of NO^*^, promoted by OER active sites provided by RuO_2_:

(14) \begin{equation*}{{\rm{N}}_2} + {}^* \to {{\rm{N}}_2}^*\end{equation*}

(15) \begin{equation*}{{\rm{N}}_2}^* +\ {\rm{O}}{{\rm{H}}^ - } \to {{\rm{N}}_2}{\rm{OH}}^* +\ {{\rm{e}}^ - }\end{equation*}

(16) \begin{equation*}{{\rm{N}}_2}{\rm{OH}}^* +\ {\rm{OH}} \to {{\rm{N}}_2}{\rm{O}}^* +\ {{\rm{H}}_2}{\rm{O}} + {{\rm{e}}^ - }\end{equation*}

(17) \begin{equation*}{{\rm{N}}_2}{\rm{O}}^* +\ {\rm{O}}{{\rm{H}}^ - } \to {{\rm{N}}_2}{\rm{O}}\!\left( {{\rm{OH}}} \right)^* +\ {{\rm{e}}^ - }\end{equation*}

(18) \begin{equation*}{{\rm{N}}_2}{\rm{O}}\!\left( {{\rm{OH}}} \right)^* +\ {\rm{O}}{{\rm{H}}^ - } \to 2{\rm{NO}}^* +\ {{\rm{H}}_2}{\rm{O}} + {{\rm{e}}^ - }\end{equation*}

This outstanding catalytic performance could be attributed to the electronic effect and spillover effect. Specifically, the upshift of the d-band center of the Ru site in TiO_2_ promoted the rate of N_2_ activation and electrochemical conversion into NO^*^ intermediates, which can combine with the active oxygen species spilled over from RuO_2_ to Ru_x_Ti_y_O_2_ to form nitrate, releasing active sites for producing more active oxygen species and accelerating the second step of NOR.

The synergetic catalytic effect for NOR was also performed on ZnFe_x_Co_2__–__x_O_4_ spinel oxides in N_2_-saturated 1 M KOH [57]. After correction, the highest yield rate of nitrate was observed to be 130 ± 12 μmol h^–1^ g_MO_^–1^ on a ZnFe_0.4_Co_1.6_O_4_ catalyst at a potential of 1.6 V (versus RHE). Nevertheless, the Faradaic efficiency is lower than that on the ZnFe_2_O_4_ catalyst, which shows the highest value of 10.1 ± 0.9% at 1.5 V (versus RHE). The higher catalytic activity towards OER will cause the lower Faradaic efficiency of NOR, meaning that with the increase in the applied potential, the rate of OER increases rapidly while the rate of NOR increases slowly. The roles of Fe and Co in ZnFe_x_Co_2__–__x_O_4_ spinel oxides on the synergetic catalytic effect were explained by DFT, suggesting that Fe could facilitate the first N–O bond formation and Co could stabilize the adsorbed OH^–^ for the further formation of the second and third N–O bonds.

**Figure 6.**
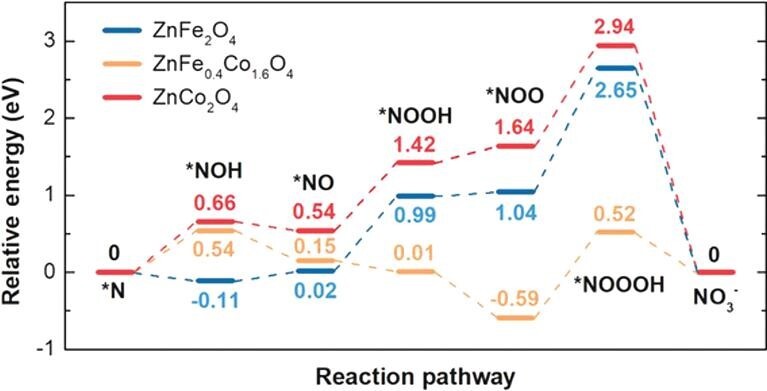
Calculated relative energy diagram of NOR processes over ZnFe_x_Co_2–x_O_4_ spinel oxides (x = 0, 0.4 and 2), reproduced with permission from Ref. [57]. Copyright 2020, Wiley-VCH.

For two completing electrochemical reactions with almost the same activation energy, the adsorption strength of the reactant on the electrode surface or adsorption energy plays a vital role because the reaction probability is primarily dependent on the amount of reactant adsorbed on the surface. However, when the reaction takes place by two different species adsorbed on the surface and one of them takes part in a competing reaction, the process becomes more complicated. In this case, the catalyst has to be well designed and synthesized to balance the adsorption and activation process for each species. For the NOR mechanism as shown in Fig. 6, it is convincing that the first N–O bond formation is a critical step. Once it is formed, further oxidation will occur relatively easily.

In acid media, Fe–SnO_2_ catalyst performed a bifunctional catalytic activity towards NOR and NRR (nitrogen reduction reaction) and NOR started from 1.6 to 2.1 V (versus RHE) [58]. For NOR, on the optimized Fe (3%)–SnO_2_ catalyst surface, the yield of nitrate and Faradaic efficiency were 42.9 μg h^–1^ mg_cat._^–1^ and 0.84% at an applied potential of 1.96 V, respectively. The yield rate was much higher than those of Ru/TiO_2_ at 2.2 V in neutral media and ZnFe_0.4_Co_1.6_O_4_ at 1.6 V in alkaline media, and the Faradaic efficiency was much lower than those of Ru/TiO_2_ at 1.8 V in neutral media and ZnFe_2_O_4_ at 1.5 V in alkaline media. The enhancement of catalytic activity for NOR on Fe–SnO_2_ was attributed to the oxygen vacancy-anchored single-atom Fe, where the energy barrier for the breakage of N≡N is lower, resulting in favorable adsorption and activation of the N_2_ molecule. It is also the reason for the improvement in NRR activity. Here, it should be considered that the catalyst, which can adsorb and activate N_2_ molecules efficiently, probably has a bifunctional catalytic activity for nitrogen fixation (NRR and NOR) in the case of the influence of the potential being negligible.

MXene, a series of novel 2D materials, was used extensively in the fields of electrochemical energy storage and catalysis. To our knowledge, the first work on using MXene-based material as an electrocatalyst for NOR was done by Yan *et al.* [59]. They found that the well-dispersed Pd on MXene performed excellent catalytic activity towards NOR in neutral media. The highest yield rate of NO_3_^–^ and Faradaic efficiency was obtained to be 2.80 μg h^–1^ mg_cat._^–1^ (or 45.16 μmol h^–1^ g_cat._^–1^) and 11.34%, respectively, at a potential of 2.03 V versus RHE corresponding to a current density of 0.4 mA cm^–2^. The process of NOR on a Pd-MXene catalyst surface was also concluded into two main steps that consist of the electrocatalytic conversion of nitrogen molecules to NO^*^ intermediates as the initial step and the non-electrochemical oxidation of NO^*^ into NO_3_^–^ as the final step. It was mentioned that OER is a competing side reaction for NOR, hindering the initial step of NOR, while appropriate O_2_ produced by OER was regarded as the reactant in the final step of NOR.

Above all, the experimental and theoretical studies showed that the onset potential of NOR is highly close to that of OER and the linear sweep voltammetry or cyclic voltammetry curves obtained in N_2_ and Ar atmospheres almost overlap, resulting in lower Faradaic efficiency. Accordingly, an efficient electrocatalyst requires sufficient active sites where the chemically inert N_2_ molecules can be selectively adsorbed and activated, and the serious parasitic OER can be effectively inhibited during N_2_ oxidation. Little progress has yet been made in developing such catalysts because there is no doubt that most of catalyst surfaces prefer to adsorb OH^–^ rather than N_2_, even if N_2_ molecules are adsorbed preferably but activated with difficulty at active sites under the specific potential. Some of them can be pushed aside by the fast adsorption of OH^–^ and the formed O_2_. Furthermore, if the appropriate OER activity may accelerate the NOR process, it is advisable to design the catalysts with two different adjacent active sites for OER and NOR, respectively. It should be noted that not all active oxygen species can combine with adsorbed N_2_ to form NO. However, most of them combine with another oxygen species to form O_2_. These suggest that the rate-limiting step for NOR is probably the effective adsorption and activation of N_2_.

To design efficient catalysts, it becomes crucial to clarify the preferential adsorption mode of N_2_ and OH^–^ at which an effective electrochemical reaction can further take place to form the first N–O bond on the catalyst surface, based on the consideration of all sorts of effects (such as electron effect, size effect, strain effect, ligand effect, boundary effect, etc.) of a well-designed catalyst. At present, bi-/multi-metallic and their oxide catalysts are attractive and promising for NOR due to the high catalytic activity for electrocatalytic reactions resulting from the synergistic effect between the different components. Consequently, it is expected that these catalysts of high activity, selectivity and stability for NOR can eventually be discovered.

### Ultrasonic conversion

Ultrasound is a kind of acoustic wave at frequencies above the audible range (above ∼20 kHz) used in cleaning, echo sounding and chemical reactions due to its good directivity and strong reflectivity. When the ultrasonic wave propagates in a medium, it undergoes physical and chemical changes due to the interaction between the ultrasound and the medium, resulting in a series of ultrasonic effects including mechanical, thermal, cavitation and chemical effects.

The ultrasonic cavitation can generate high local instantaneous temperatures and pressures and sonoluminescence [60,61]. Moreover, radicals generated during the cavitation can induce chemical reactions—the so-called chemical effects of ultrasound. These complex effects are not yet thoroughly clarified. Nevertheless, some theoretical models have been established to describe the origins of molecular activation. It was found that the molecules at the interior of the bubble of cavitation filled with vapor and gas are excited and further dissociated [62–67]. Inside the bubbles or at the interface of the two phases, the generated radicals can combine with gas to form products. Nitrogen oxidized to nitrite and nitrate directly have been achieved in aqueous medium saturated with air under an ultrasonic field.

As early as 1936, Gohr *et al.* found that H_2_O_2_, HNO_2_ and HNO_3_ were generated in water saturated with air under an ultrasonic field at a frequency of 540 kHz and the HNO_3_ formation is due to the further oxidation of HNO_2_ when oxygen is sufficient in water [68]_._ Subsequently, further investigations of nitrogen fixation in the ultrasonic field were launched. In 1950, Ellfolk *et al.* [69,70] explored the factors affecting the oxidative nitrogen fixation in the ultrasonic field and found that the ratio of nitrite to nitrate was determined by the hydrogen ion concentration (or pH) of the solution and the formed H_2_O_2_ was lessened rapidly at a pH of <4. The authors considered that it is due to the consumption of H_2_O_2_ to oxidize nitrite to nitrate rather than the diminishing of the formation of H_2_O_2_. Furthermore, it was found that the process of nitrogen fixation in the ultrasonic field was inhibited in the presence of hydrogen and carbon monoxide, probably as a result of the competition of hydrogen and nitrogen for oxygen. Finally, based on the point of view of ionization potential, the first activation step of the aerobic fixation of nitrogen in the ultrasonic field was discussed and the same possible reaction pathways were summarized, as follows:

Possible reaction pathway 1:

(19) \begin{equation*}{\rm{N}}_2^ + + {\rm{\ }}{{\rm{O}}_2} = {\rm{\ }}2{\rm{NO\quad N}}_2^ + + {\rm{\ }}{{\rm{O}}_2} = {\rm{\ NO}}_2^ + + {\rm{N}} \!\bullet \end{equation*}

(20) \begin{equation*}{{\rm{N}}^ + } + {\rm{\ }}{{\rm{O}}_2} = {\rm{\ N}}{{\rm{O}}_2}{\rm{\quad NO}}_2^ + + {\rm{\ O}}_2^ - = {\rm{\ N}}{{\rm{O}}_2} + {{\rm{O}}_2}\end{equation*}

(21) \begin{equation*}{\rm{N}}_2^ + + {\rm{O}} \!\bullet {\rm{\ }} = {{\rm{N}}_2}{\rm{\ O\quad N}}_2^ + + 2{\rm{\ }}{{\rm{O}}_2} = {\rm{\ }}{({{\rm{N}}_2}{{\rm{O}}_4})^ + }\end{equation*}

(22) \begin{equation*}{{\rm{N}}^ + } + {\rm{O}} \!\bullet {\rm{\ }} = {\rm{\ NO\quad }}{({{\rm{N}}_2}{{\rm{O}}_4})^ + } + {\rm{e\ }} = {\rm{\ }}2{\rm{N}}{{\rm{O}}_2}\end{equation*}

Possible reaction pathway 2:

(23) \begin{equation*}{{\rm{N}}_2} \to {\rm{N}} \!\bullet \end{equation*}

(24) \begin{equation*}{{\rm{O}}_2} \to {\rm{O}} \!\bullet \end{equation*}

(25) \begin{equation*}{\rm{N}} \!\bullet +\ {\rm{O}} \!\bullet {\rm{\ }} \to {\rm{NO}}\end{equation*}

Verrall *et al.* studied the variety and yield of ultrasonic products in water in the presence of dissolved gases [71]. The results indicated that the amounts of the formed hydrogen peroxide and total nitrogen fixation depend on the nature of the dissolved gases. For hydrogen peroxide, the formation follows the order O_2_ > air > Ar > N_2_. However, it follows the order air > N_2_ > Ar > O_2_ for the total amount of nitrogen fixation. In the case of using 447 kHz at 50 W irradiating, the initial formation rates of nitrite and nitrate in water saturated with air at 298 K were 22 × 10^–9^ and 6 × 10^–9^ mol min^–1^ W^–1^, respectively. The authors proposed that the aerobic fixation of N_2_ undergoes the dissociation of nitrogen molecular and oxygen molecular to atoms and then atomic nitrogen and atomic oxygen combined to form nitric oxide. In the absence of oxygen, the formed atomic nitrogen is reacted with hydroxyl radicals to produce NOH intermediates, which can further combine with hydroxyl or HO_2_ radicals to form nitric oxide and water or hydrogen peroxide.

However, it was also suggested that the presence of nitrous and nitric acid in the absence of oxygen in water is probably attributed to either the impurity in the dissolved gases or residual air in the incompletely degassed water. Activation of nitrogen by •OH radicals and atomic oxygen and further oxidation processes were suggested by Petrier, as follows [72]:

(26) \begin{equation*}\bullet\! {\rm{OH}} + {{\rm{N}}_2} \to {{\rm{N}}_2}{\rm{O}} \to {\rm{NO}}_2^ - \to {\rm{NO}}_3^ - \end{equation*}

(27) \begin{eqnarray*}&&{\rm{O}} \bullet +\ {{\rm{N}}_2} \to {{\rm{N}}_2}{\rm{O}},\quad {\rm{\ NO}},\\ && {\rm{\ N}} \!\bullet {\rm{\ }} \to {\rm{NO}}_2^ - \to {\rm{NO}}_3^ - \end{eqnarray*}

On the contrary, it was also reported that the •OH radicals arising secondarily from water are evidently unable to oxidize nitrogen [70].

Ultrasonic frequency dependence of the yields of nitrite and nitrate in air-saturated water has been investigated by Tiehm *et al.* [73]. In the range of 41–3217 kHz, the maximum yields were obtained at 360 kHz, which gives the formation rates of 7.1 mg (as N) L^–1^ for nitrate and 0.6 mg (as N) L^–1^ for nitrite, corresponding to 42 × 10^–9^ and 4 × 10^–9^ mol min^–1^ W^–1^, respectively. In the same report, the total nitrate plus nitrite formation rate of 33 × 10^–9^ mol min^–1^ W^–1^ was obtained by using 30 W of 500 kHz ultrasound in 200 mL air-saturated water at 293 K by Petrier *et al.* [73]. Besides, it was found that the yield of products changed with the irradiating time. The rate of nitrate formation increased steadily, while nitrite decreased after 100 min. The hydrogen peroxide formation rate was initially about the same as the total nitrate plus nitrite but decreased after 100 min. Therefore, it was considered that the primary products are hydrogen peroxide and nitrite, and then nitrate was formed via a pH-dependent oxidation reaction of nitrite by hydrogen peroxide.

Subsequently, Kruus *et al.* [74] investigated the effect of time, temperature and gas composition on the nitrite- and nitrate-formation rate in 100 mL air-saturated water at 278 ± 2 K under 27 W of 900 kHz ultrasound irradiation. The total formation rate increased with the decrease in temperature and with the increase in O_2_ fraction up to between 0.4 to 0.5 and then decreased. The highest formation rate of total nitrate plus nitrite over 20 min was 16 × 10^–5^ M, which is equivalent to 30 × 10^–9^ mol min^–1^ W^–1^ and close to the values presented above. Recently, Kobayashi *et al.* compared the yields of nitrite and nitrate in air, O_2_, N_2_ and Ar-saturated ultrapure water under a 23-, 28- and 43-kHz ultrasound field with 200–1200 W of output power. The optimum frequency was found to be 28 kHz and the higher the power supplied, the higher the yields produced. In these optimized conditions, the formation rate of nitrite and nitrate was deduced to be ∼0.60 and 0.44 μM min^–1^, respectively. However, in the presence of N_2_, O_2_ and Ar, the yields of these products are very low compared to those in the atmosphere, as Mead observed before.

To improve the nitrogen-conversion efficiency in gas-saturated aqueous solution under the ultrasound field, the idea should be guided to enhance the cavitation effect, the yield and stability of active radicals, gas solubility and dissolved fraction, and active sites of a catalyst. There are several effect factors behind this, such as pH, ionic strength, frequency, power density, gas composition, temperature, catalyst, etc. In the future, the influence rules and mechanisms of each effect factor among them should be elucidated. Based on this knowledge, it is easy to find an optimal solution for nitrogen fixation by ultrasound. For the catalyst, thermoelectric and piezoelectric materials should be given priority because these materials may exhibit excellent polarization performance under high pressure and high temperature produced by ultrasound.

### Photon-driven conversion

**Figure 7.**
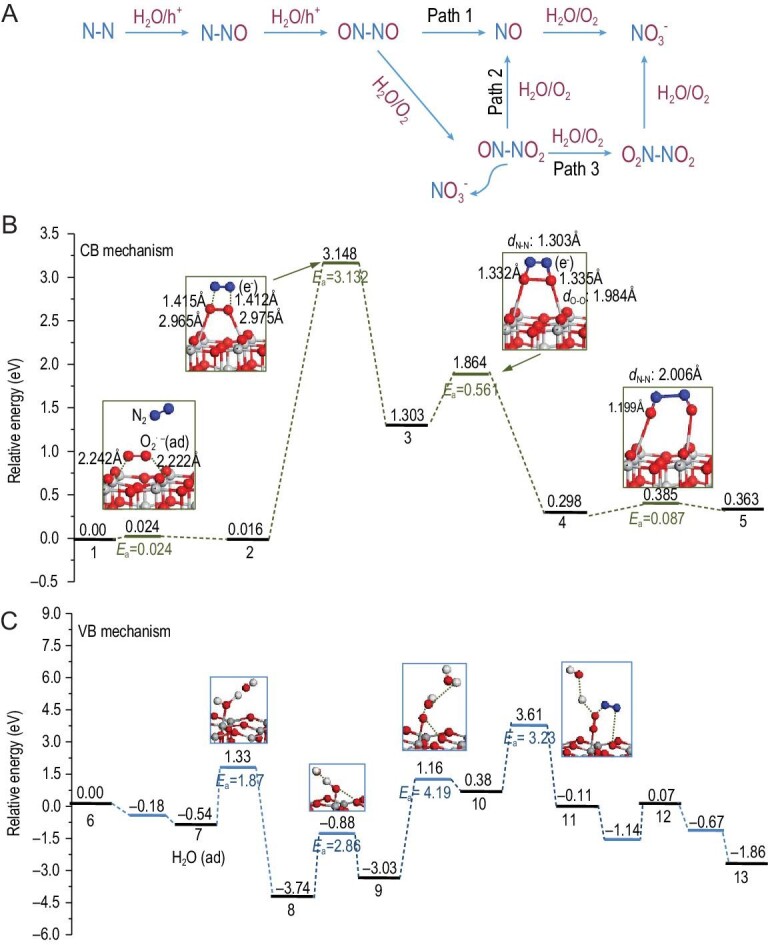
(A) Routes for photo-oxidation of the nitrogen reaction mechanism, reproduced with permission from Ref. [75]. Copyright 2019, Royal Society of Chemistry. (B) and (C) Calculated potential energy profile of each step for NO formation on the conduction band and the valence band of TiO_2_ (001) surface, respectively, reproduced with permission from Refs [76,77]. Copyright 2013, Springer Nature.

Photocatalytic conversion of N_2_ with O_2_ represents a practically viable oxidative conversion of air not driven by fossil fuels. The photocatalytic nitrogen oxidation process can be divided into several steps: first, excitation of electrons from the valence band (VB) to the conduction band is initiated by light absorption, leaving holes in the VB. Then the photo-generated h^+^ oxidizes N_2_ to NO with water, while O_2_ is reduced to H_2_O by photoexcited electrons, and NO is further oxidized to nitrates evolving O_2_ and H_2_O, as shown in Fig. 7A. Overall, nitrate acid is synthesized from water, O_2_ and N_2_ under ambient conditions using sunlight as an energy source [75].

Photocatalysis as a green, renewable and sustainable technique has attracted massive attention on activating N–N bonds using various photocatalysts such as Diamond [78], BrOX [79,80], Mo-doped W_18_O_49_ [81], Bi_5_O_7_Br [82,83], TiO_2_ [84], LDH [85,86] and g-C_3_N_4_ [87]. In this field, studies have mainly focused on nitrogen fixation to NH_3_ products, while a reaction involving N_2_ with O_2_ has been rarely attempted. In 2013, Yu *et al.* first reported a direct nitrate-formation process from atmospheric nitrogen and oxygen on nano-sized TiO_2_ surfaces under UV or sunlight irradiation [76,77]. This work is of significance as the group demonstrated that a continuous nitrate-producing reaction was observed over time. They detected an intermediate gaseous product at a retention time of 1.25 min by comparing with the results of the gas chromatogram before and after the photocatalytic reaction. According to the results of Fourier transform infrared (FTIR) differential spectrum and theoretical calculations (as shown in Fig. 7B and C) for NO formation, it was suggested that NO is an intermediate product. Afterward, Zhang *et al.* [88] successfully used Z scheme heterojunction TiO_2_/WO_3_ nanorods as a photocatalyst to synthesize NO, which is an intermediate product in photocatalytic nitrogen oxidation, and its production rate was determined to be 0.16 mmol g^–1^ h^–1^ associated with thermal energy (at 300°C) and quantum efficiency of 0.31% at 365 nm.

Photocatalytic nitrogen activation and oxidation were achieved at the photo-generated holes on the VB of semiconductors. Therefore, photocatalysts containing abundant potholes were beneficial to nitrogen fixation to nitrate. Recently, Xie *et al.* reported that pothole-rich WO_3_ nanosheets can activate the N≡N bond and synthesize nitrate directly under ambient conditions [89]. Pothole-rich WO_3_ exhibited an efficient photocatalytic performance. The average rate of nitrate production is as high as 1.92 mg g^–1^ h^–1^ under ambient conditions, without any sacrificial agent or precious-metal co-catalysts under UV/Vis irradiation at 380 nm. The apparent quantum efficiency (AQE) was calculated to be 0.11%, which is better than pothole-free nanosheets and bulk WO_3_.

Photoconversion of nitrogen to nitrate under ambient conditions is expected as an alternative cost-effective approach for producing nitrate. However, the rate and AQE of nitrate production are too slow to meet the demand of industrial production. Great endeavors are essential to increase the efficiency of nitrate production. Moreover, the existing studies demonstrated that a good synergy between photon energy and thermal energy is more beneficial to nitrogen-conversion reactions. The development of an innovative catalytic process associated with thermal energy, or even another energy input, exhibits great potential to achieve efficient nitrogen conversion.

## N_2_ + O_2_ + X

As discussed above, the direct oxidation of N_2_ is rather challenging due to the high energy threshold. Introducing another chemically active molecule ‘X’ alone with air to modify the reaction pathway, thus undergoing a relatively lower energy potential, could be another method to achieve the conversion of air. Moreover, the selective synthesis of high-value C–N–O organics from key components of air and C-containing ‘X’ is a holy grail in chemistry.

Inert gas (active to plasma) has been widely applied in plasma conversion of N_2_ and O_2_, mainly participating as a third-body molecule and stabilizing the discharge. Therefore, we do not include inert gas in the category of X gas in the NTPs process. Some other studies have investigated the plasma-driven reactions between CH_4_ and air [90] and simulation on plasma conversion of a gas mixture of CH_4_/CO_2_/N_2_/O_2_ [91]. Even though these studies took a brief glimpse into the plasma chemistry of the conversion of N_2_, the main goal is still the conversion of CH_4_ and/or CO_2_, and needless to say the underlying reaction pathway and the possibility to soften the high energy barrier. H_2_O has also been involved in the NTP conversion of N_2_/O_2_ [39]. The results indicate that the atomic oxygen and hydroxyl radical (OH) generated from O_2_ and H_2_O significantly affect the formation of NO_x_, proving that the presence of H_2_O enhanced the conversion of N_2_ and formation of NO_x_ rather than N_2_/O_2_ [39]. The addition of NO was also studied by some groups [39,40]. However, these works emphasized the oxidation of NO into NO_2_ and little information on the conversion of N_2_ with O_2_ can be found. Based on these existing works, further investigation should be made on how the third molecule ‘X’ mitigates the energy consumption for the conversion of N_2_ with O_2_. We believe that involving a chemically active molecule ‘X’ can be a feasible pathway in the NTP conversion of N_2_ with O_2_ and this pathway needs tremendous endeavors on both exploring an appropriate ‘X’ with appropriate catalysts and the underlying chemistry.

While for electrochemical conversion, it seems a great challenge to achieve the electrochemical conversion of N_2_, O_2_ with ‘X’ (such as CH_4_, CO_2_ or organic molecules) into organic compounds consisting of C–N–O (such as CH_4_N_2_O, RNH_2_, RCHNH_2_COOH, RNO_2_) because of the simultaneous adsorption and activation of N_2_, O_2_ with ‘X’ at the same or neighbor active sites under the same conditions, is almost impossible not to mention the bonding that includes the oxidation and reduction reactions simultaneously. However, it can become possible when the whole process sequentially passes through electron-transfer steps and chemical steps. More precisely speaking, the active species produced electrochemically at the active sites on the catalyst surface can induce further electron transfer or chemical reaction with other reactants nearby to form another active intermediate, which will take a further electrochemical or chemical reaction with the third reactants at the active site or in the electrolyte to form the final products, mimicking a chain reaction. According to the existing studies, the coupling reaction of N–O–X should be started with the activation of O_2_ to form superoxides and peroxides, which are expected to trigger this chain reaction.

Ultrasound induction could also be a possible method to achieve the coupling reaction of N_2_/O_2_/‘X’ due to its cavitation effect and chemical effect, which could dissociate N_2_, O_2_, X and H_2_O into atoms and/or active species, leading to the occurrence of some chemical reactions between multiple species. Particularly if the introduced ‘X’ molecule is chemically active under ultrasound conditions in the presence of catalysts, it probably enhances the conversion of N_2_ and O_2_ with lower energy potential. Even though no studies on this strategy have been reported, ultrasound induction is adopted widely in the field of organic synthesis involving N_2_ bonds with various components. Similar to plasma-driven or ultrasonic conversion, some active species and radicals can be produced during the photocatalytic process and the category and number of these species depend mainly on the solvent and the surface performance of a photocatalyst.

**Scheme 2.**
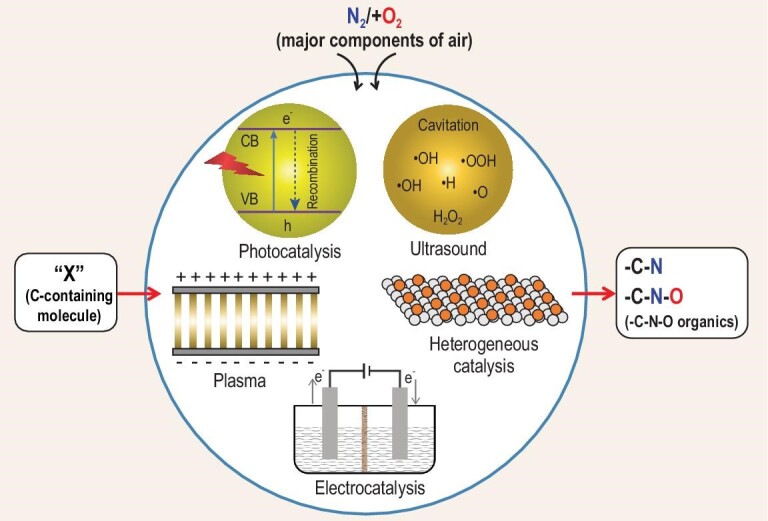
The scheme of the conversion of N_2_/O_2_ with ‘X’ via multiple processes.

Reasonably coupling two or more aforementioned processes (as shown in Scheme 2) can provide great potential to effectively transform N_2_, O_2_ and ‘X’ into high-value C–N–O organics as the reaction coordinates may differ greatly from one to another. For example, thermal catalysis undergoes the chemical reaction via a translational mode, while plasma catalysis initiates the chemical reaction by electron impact and vibrational and electronic excitation. It is worth exploring both the fundamental science of the possible synergies and the engineering improvements to amplify the synergies.

## FUTURE PERSPECTIVE

Since the beginning of the twentieth century, the demand for nitrogen fixation has been dramatically increasing. Researchers used to commercialize the B–E process to produce NO_x_ from the air, yet it was soon surpassed by the H–B process due to the higher energy efficiency. However, this process still suffers from extreme conditions, consuming pure hydrogen sources and generating ∼1.9 metric tons of CO_2_ per metric ton of NH_3_ production. The one-step direct conversion of air for NO_x_ products rather than the H–B process coupled with the Ostwald process is more attractive in terms of theoretical energy efficiency, enrichment of feedstock and the convenience for engineering design if it can be successfully realized. The most severe challenge for this process is to overcome the tremendous-amount-of-energy barrier. Therefore, a sole thermochemical or thermal plasma process is not favorable as few catalysts are stable in such high temperatures. Other processes, such as non-thermal plasma, electrochemical, ultrasonic and photon-driven conversion, could be appropriate to convert air into desired NO_x_ products under soft conditions. However, not many efforts have been dedicated to this challenging field compared with ammonia synthesis. To make a brief conclusion of existing works, great opportunities exist in the direct conversion of N_2_ with O_2_, while progress will require far more improved energy efficiency (at least <3000 kJ mol^–1^ N_2_) from a macro perspective, a molecular-level understanding of nitrogen transformation reactions, as well as mechanistic insights into the discovery of new catalytic systems and multiple means of delivering the energy needed to drive those reactions from a micro perspective.

What is important for the future? This review has addressed the technical and scientific challenges of the direct conversion of air into NO_x_ products. The authors also would like to provide some perspectives and strategies, some of which have been briefly discussed, for further investigation on the direct conversion of N_2_ and O_2_: (i) coupling multiple processes, (ii) introducing another gas molecule to undergo a softer reaction pathway and (iii) development of new catalysts.

Multiple processes of coupling allow multiple forms of energy input, thus providing different pathways to activate N_2_ and trigger its reaction. A sole thermochemical process suffers from the extreme conditions, high requirements for equipment and low energy efficiency. Coupling thermochemical process with the other energy inputs is simply designed and has been applied in thermo-electro, thermo-photo and thermo-plasma conversion as we discussed above. The enhancement on the conversion of N_2_ and O_2_ may be significantly amplified with the other energy inputs by tuning different reaction coordination. For example, a conversion of 3.8% and energy efficiency of 2000 kJ mol^–1^ for N_2_ can be achieved in a microwave plasma reactor by coupling thermal and plasma processes [30]. Once the benefit of energy efficiency surpasses the cost for reaching those conditions, it will be feasible for future scalability.

On the other hand, the conversion of N_2_ and O_2_ under mild conditions is the goal for the scientific community, which is also the focus of this review. The coupling of two or more processes among plasma-chemical, electrochemical, ultrasonic and photon-driven conversion can be one of the promising solutions to achieving this goal due to their special methods of energy inputs. However, no attempts at systematic studies have been made to find an effective method or process for coupling multiple energy inputs and investigating the possible synergy between those. Tremendous fundamental studies are required to discover how the synergies between different processes and catalysis work, and to realize sophisticated engineering improvements to maximize the synergies.

Direct conversion of N_2_, O_2_ and C-containing ‘X’ into high-value C–N–O organics is the long-standing and final pursuit of key components of air transformation. Although no studies have been reported to our knowledge until now, introducing a chemically active ‘X’ can alter the reaction pathways, thus probably undergoing lower energy thresholds for which some works have demonstrated the possibility. Exploration has been conducted on seeking an appropriate ‘X’ for different processes. It also requires extensive studies and in-depth comprehension of fundamental chemical coordination. The development of a new catalytic system and the exploration of the catalytic mechanism is also one of the permanent research cores for all these processes, particularly for coupling processes. The understanding of catalysis under this complex system is of great importance for catalyst design. All these advances will emerge through collective understanding and insights to be comprehended from fundamental research that associates experiments and theory in catalysis and different processes.
